# Rainfall and sentinel chicken seroconversions predict human cases of Murray Valley encephalitis in the north of Western Australia

**DOI:** 10.1186/s12879-014-0672-3

**Published:** 2014-12-10

**Authors:** Linda A Selvey, Cheryl A Johansen, Annette K Broom, Catarina Antão, Michael D Lindsay, John S Mackenzie, David W Smith

**Affiliations:** School of Public Health, Curtin University, Perth, 6845 WA Australia; The Arbovirus Surveillance and Research Laboratory, M504 School of Pathology and Laboratory Medicine, QEII Medical Centre, The University of Western Australia, Nedlands, 6009 WA Australia; PathWest Laboratory Medicine, QEII Medical Centre, Nedlands, 6009 WA Australia; NSW Department of Family and Community Services, 320 Liverpool Rd, Ashfield, 2131 NSW Australia; Environmental Health Directorate, WA Health, Perth, WA Australia; Faculty of Health Sciences, Curtin University, Perth, 6845 WA Australia; School of Pathology and Laboratory Medicine, Faculty of Medicine, Dentistry and Health Sciences, University of Western Australia, Perth, WA Australia

**Keywords:** Murray valley encephalitis, Sentinel chicken surveillance, Epidemiology, Human risk, Flavivirus, Environmental factors

## Abstract

**Background:**

Murray Valley encephalitis virus (MVEV) is a flavivirus that occurs in Australia and New Guinea. While clinical cases are uncommon, MVEV can cause severe encephalitis with high mortality. Sentinel chicken surveillance is used at many sites around Australia to provide an early warning system for risk of human infection in areas that have low population density and geographical remoteness. MVEV in Western Australia occurs in areas of low population density and geographical remoteness, resulting in logistical challenges with surveillance systems and few human cases. While epidemiological data has suggested an association between rainfall and MVEV activity in outbreak years, it has not been quantified, and the association between rainfall and sporadic cases is less clear. In this study we analysed 22 years of sentinel chicken and human case data from Western Australia in order to evaluate the effectiveness of sentinel chicken surveillance for MVEV and assess the association between rainfall and MVEV activity.

**Methods:**

Sentinel chicken seroconversion, human case and rainfall data from the Kimberley and Pilbara regions of Western Australia from 1990 to 2011 were analysed using negative binomial regression. Sentinel chicken seroconversion and human cases were used as dependent variables in the model. The model was then tested against sentinel chicken and rainfall data from 2012 and 2013.

**Results:**

Sentinel chicken seroconversion preceded all human cases except two in March 1993. Rainfall in the prior three months was significantly associated with both sentinel chicken seroconversion and human cases across the regions of interest. Sentinel chicken seroconversion was also predictive of human cases in the models. The model predicted sentinel chicken seroconversion in the Kimberley but not in the Pilbara, where seroconversions early in 2012 were not predicted. The latter may be due to localised MVEV activity in isolated foci at dams, which do not reflect broader virus activity in the region.

**Conclusions:**

We showed that rainfall and sentinel chickens provide a useful early warning of MVEV risk to humans across endemic and epidemic areas, and that a combination of the two indicators improves the ability to assess MVEV risk and inform risk management measures.

**Electronic supplementary material:**

The online version of this article (doi:10.1186/s12879-014-0672-3) contains supplementary material, which is available to authorized users.

## Background

Murray Valley encephalitis virus (MVEV) is a flavivirus in the Japanese encephalitis serological complex [1]. It occurs in Australia and on the island of New Guinea, and probably on islands in the eastern Indonesian archipelago [1],[2]. The virus is particularly active in the remote Kimberley region of northern Western Australia (WA), where it is considered to be enzootic [1]. *Culex annulirostris* mosquitoes are the major vectors of MVEV, and waterbirds, particularly ardeids, are thought to be the main vertebrate host. *Aedes* mosquitoes, particularly *Aedes normanensis* and *Aedes tremulus* may also play a role in transmission as MVEV has been isolated from these species [3],[4], and they are opportunistic feeders on birds [5],[6]. MVEV spread in northern and central Australia is believed to be via movement of these birds into areas of flooding following heavy rainfall [2], though there is also evidence that the virus can survive across dry seasons in desiccation-resistant mosquito eggs in enzootic areas [3]. MVEV infection is usually asymptomatic or causes a mild febrile illness, but 1:150 to 1:1000 infected individuals develop a severe meningoencephalitis with a mortality of 20-25% and a high rate of residual neurological disease [7].

Surveillance of MVEV activity throughout the year is important in order to detect increased risk as early as possible and provide appropriate advice to the public about that risk and about mosquito avoidance. Trapping and testing of mosquitoes is difficult in sparsely populated areas of central and northern Australia, especially during the highest risk periods when flooding prevents vehicular access to large areas. In Western Australia, mosquito surveys are performed annually in major towns and communities in the Kimberley region and opportunistically in the Pilbara late in the wet season, but data from these surveys are insufficient to predict risk of MVEV infection. To overcome these problems in Australia, sentinel chickens have been successfully employed for surveillance of MVEV since 1973 [8],[9], and in WA since 1979. They are used because the vector species feed on them, they asymptomatically seroconvert following MVEV infection, and they develop a low level viraemia, limiting the likelihood that they would act as amplifying hosts for the virus [10],[11].

Six sentinel chicken flocks were established in WA by 1981, and this rose to 24 flocks by 1990 [12], and 29 flocks by 2001 [13]. Initially the flocks were located at a number of towns in the Kimberley and Pilbara regions, then were extended to the Gascoyne and Mid-West regions following MVEV activity in those regions in 1997 and 2000 [14]. Sampling was intermittent before about 1985, after which regular sampling regimens were established.

The sentinel chicken surveillance is also used to monitor activity of the Kunjin strain of West Nile virus (WNV_KUN_), the other flavivirus of major human health concern in these areas. However, diagnosed human infection is rare so we were unable to evaluate this aspect of the surveillance system.

MVEV activity occurs in areas of WA with very low population density. To provide some perspective on this, the approximate populations of the major centres in the area are: Kununurra (7,000), Broome (12,700), Port Hedland (15,000), Karratha (16,500), and Exmouth (2,200). However, there is also significant transient population in these areas for tourism and mining [15].

It is known that MVEV activity and human disease in northern and central Australia is more likely in years of heavy wet season rains and extensive flooding [14],[16]. The relationship between MVEV activity and exceptional rainfall has also been recognised in eastern Australia [17],[18]. A large outbreak of human cases of MVEV infection in WA and the Northern Territory in 2000 was associated with very high rainfall [14],[19], as was an even larger outbreak in 2011 that included human cases in WA, the Northern Territory, South Australia and New South Wales [15].

However, rainfall is not always followed by MVEV activity and the level of activity and the risk to human health also varies from season-to-season. The intent of sentinel chicken surveillance is to be able to increase the reliability of the prediction of increased risk of human disease due to MVEV infection within a time frame that will allow appropriate public health mitigation strategies [15]. A number of factors may influence the effectiveness and value of sentinel chicken surveillance [20], so it is important to evaluate individual programs. However the relatively small number of human cases of MVEV disease and their irregular occurrence is a challenge and requires accumulation of data over a long period of time.

This paper analyses the relationship between rainfall, detection of MVEV antibodies in sentinel chickens, and human cases of encephalitic and identified non-encephalitic MVEV disease over a 22 year period in WA, in order to define predisposing factors for MVEV activity, increase our understanding of MVEV ecology, and improve the predictive capability of the MVEV surveillance program.

## Methods

### Sentinel chickens

Details of WA sentinel chicken programs for MVEV surveillance have been described elsewhere [13],[21]. In WA, each flock consists of 12 chickens that are replaced annually in September-October prior to the usual wet season December-June. Additional replacement chickens are sent to locations when more than six chickens in one flock seroconvert to MVEV, WNV_KUN_ or another flavivirus, died or went missing during the high-risk season for flavivirus activity. As a rule, all chickens in each flock were bled approximately fortnightly between December and June. Compliance with the bleeding schedule varied over time and location depending on local logistics. For the purposes of this study, sentinel chicken data were compiled monthly to coincide with monthly rainfall data. Twelve sentinel chicken sites were excluded from the analysis because of inconsistent data or because testing continued for a limited number of years only.

Chicken sera were tested for the presence of flavivirus antibodies using an epitope-blocking enzyme immunoassay with the monoclonal antibody 3H6 [22]. Positive samples were then tested in similar epitope blocking enzyme immunoassays for MVEV and WNV_KUN_ antibodies using monoclonal antibodies 10C6 and 31112G, respectively [22]. Inhibition ≥50% was regarded as positive in all assays. Since 2009, samples considered positive to both MVEV and WNV_KUN_ have been titrated in order to determine the specificity of infection. Samples were deemed positive to a single virus if they had at least a four-fold greater antibody titre to the one, otherwise they were called positive for both.

Where there was at least one chicken seroconversion in a month and where there had been no sentinel chicken testing for two or more months directly prior to that seroconversion, the exact month of seroconversion was unknown. In this case, the month of seroconversion was inferred to be the midpoint of the previous months when no testing occurred. This was the case on three occasions only.

The sentinel chicken surveillance program was approved by the UWA Animal Ethics Committee (RA/3/100/1122).

### Human cases

Human cases were identified by laboratory testing following clinical presentation with symptoms. The majority of human cases had neuroinvasive infections. Testing for all suspected human cases was performed at PathWest Laboratory Medicine WA at the QEII Medical Centre (PathWest). Data (place and date of onset) on the human cases were compiled from published information [13]-[15],[23] and PathWest records and were cross-checked with the Western Australian Notifiable Infectious Diseases Database. Murray Valley encephalitis is a Notifiable disease in WA, and this study involved using data acquired through routine follow up. Further analysis of the data as was performed in this study forms part of the investigation of a Notifiable disease and as such neither Human Research Ethics Committee approval or patient consent were necessary.

### Rainfall data

**Figure 1:**
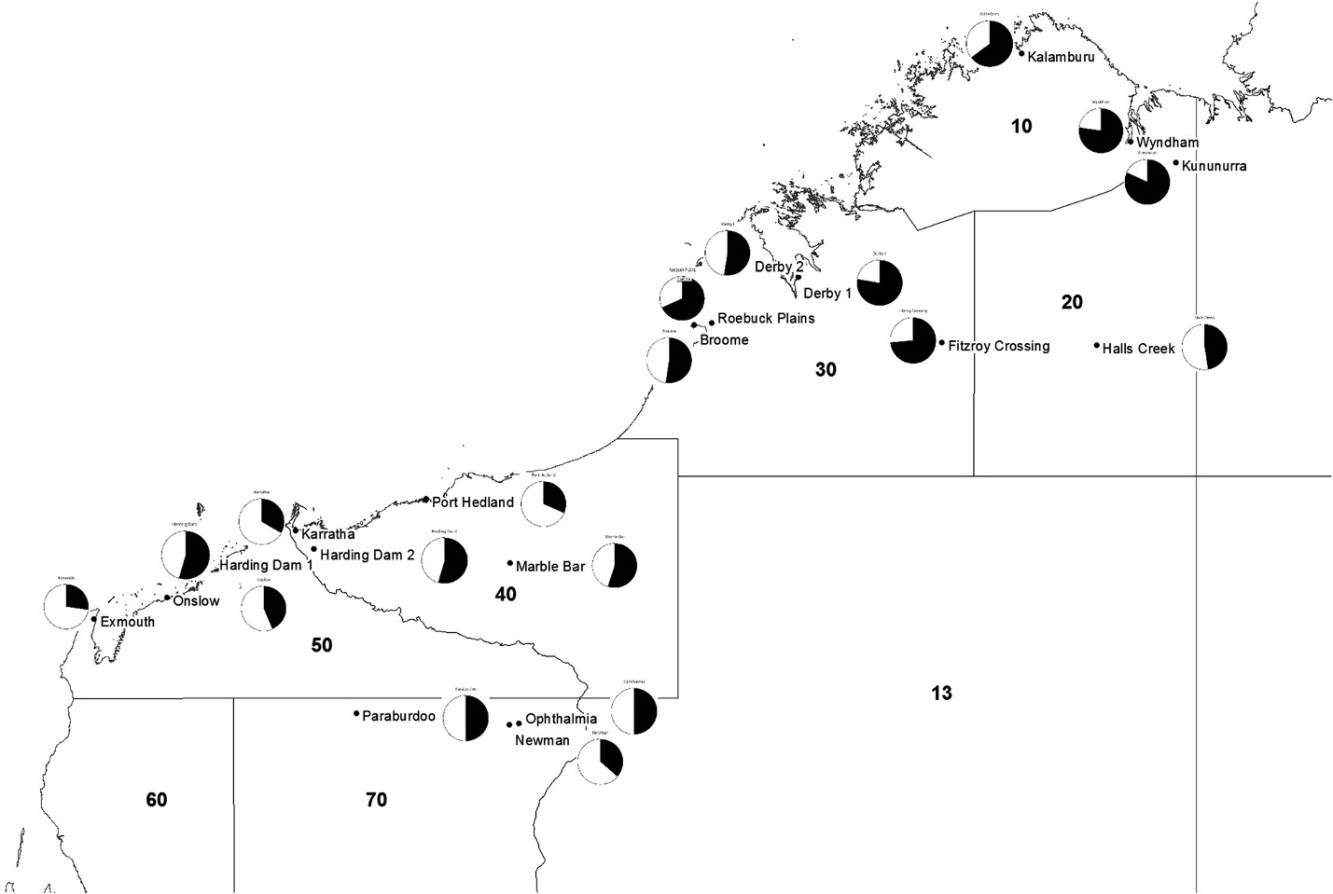
**Map of sentinel chicken sites in the study area.** Map of sentinel chicken sites in the study area with sufficiently consistent testing to be included in the data analysis by rainfall districts. The pie charts show the proportion of the years in which chickens tested positive compared to the years that testing occurred. (Note that the sentinel chicken testing program also includes sites south of the study area, but these were not included in the analysis).

Monthly district rainfall data were obtained from the Bureau of Meteorology (BOM) [24]. We used these data in preference to averaging the monthly rainfall for each site because of difficulties with inconsistency of rainfall recording at each site over time. The BOM rainfall districts were defined for the purpose of recording rainfall only and do not coincide with any administrative boundaries [25]. There was a strong correlation between the BOM’s monthly rainfall district data and the monthly rainfall averaged across each sentinel chicken testing site in that district (Pearsons Correlation Coefficient >0.900 for all rainfall districts other than District 40, where it was 0.887, p < 0.001 for all Districts). Three sites, Ophthalmia Dam, Paraburdoo and Newman were included in District 50 for the purpose of this analysis as, while they are actually situated in rainfall District 70, they are close to the border with District 50 (Figure 1). There is a strong correlation between the monthly rainfall data in District 70 and District 50 (Pearsons Correlation Coefficient R = 0.907, p < 0.001).

### Statistical analysis

Sentinel chicken and human case data from June 1990 to December 2011 were included in the analysis. We used a negative binomial regression model to identify factors associated with the numbers of infected chickens (offset by the total number of chickens tested), and a similar model for the number of human cases identified. The independent variables included monthly rainfall data from the previous three months, proportion of seropositive chickens in the previous month, and season. Models were developed separately within each rainfall district, using a backwards elimination strategy. In this strategy, all independent variables were initially included in the model, then the least significant was dropped (one at a time), until all variables remaining in the model were significantly associated with the outcome (p < 0.05). We tested the chicken model on the 2012 and 2013 rainfall and sentinel chicken data by comparing actual chicken seroconversion data and predicted model outcomes, as assessed using the following formula:

$$\mathrm{Seroconversion}=Exp\left[\mathrm{Intercept}+\left(b1*X1\right)+\left(b2*X2\right)+\left(b3*X3\right)\ldots .\right]\text{,}$$

where b*n* and X*n* represent the estimated parameters and the independent variables in the model, respectively.

All analyses were performed using the SPSS version 21 statistical software, and, following convention, a p-value < 0.05 was taken to indicate a statistically significant association in all tests.

## Results

### Sentinel chicken data

Sentinel chicken sites in the Kimberley (Districts 10, 20 and 30) and Pilbara (Districts 40, 50 and part of 70) that provided samples regularly were included in the analysis. All sites tested chickens from July 1990 onwards, with the exception of a second site in Derby (1993 onwards), Fitzroy Crossing (1993 onwards) and Onslow (1994 onwards) (Figure 1). The number of chickens tested each month at each site varied, depending on the availability of chickens and whether chickens had tested positive in the previous month and therefore were excluded. On average, eleven chickens were tested at each site per month (range 0 – 54).

Overall, the proportion of years when at least one seroconversion was observed at each sentinel site varied from 48% (Halls Creek) to 83% (Kununurra) in the Kimberley and 30% (Exmouth) to 57% (Harding Dam and Marble Bar) in the Pilbara (Figure 1). The proportion of chickens testing positive also varied between sites and months tested from a low of zero to a high of 100%.

Chicken seroconversion in one or more sites in the Kimberley preceded seroconversion in Districts in the Pilbara by at least one month except in March 1997, January 2000, and February 2009. The sites in the Pilbara where chicken seroconversion coincided with seroconversion in the Kimberley were Paraburdoo, Ophthalmia, and/or Harding Dam. In those years (1997, 2000 and 2009), there were large numbers of chicken seroconversions (>100) across all Districts, suggesting high levels of MVEV activity. Chicken seroconversion occurred in the Kimberley but not in the Pilbara in 1998, 2005, 2007, and 2010. In 2005, the only site where seroconversions occurred was Kununurra. There were no seroconversions at all in 1990 (July to December) and 1996.

### Human cases

**Figure 2:**
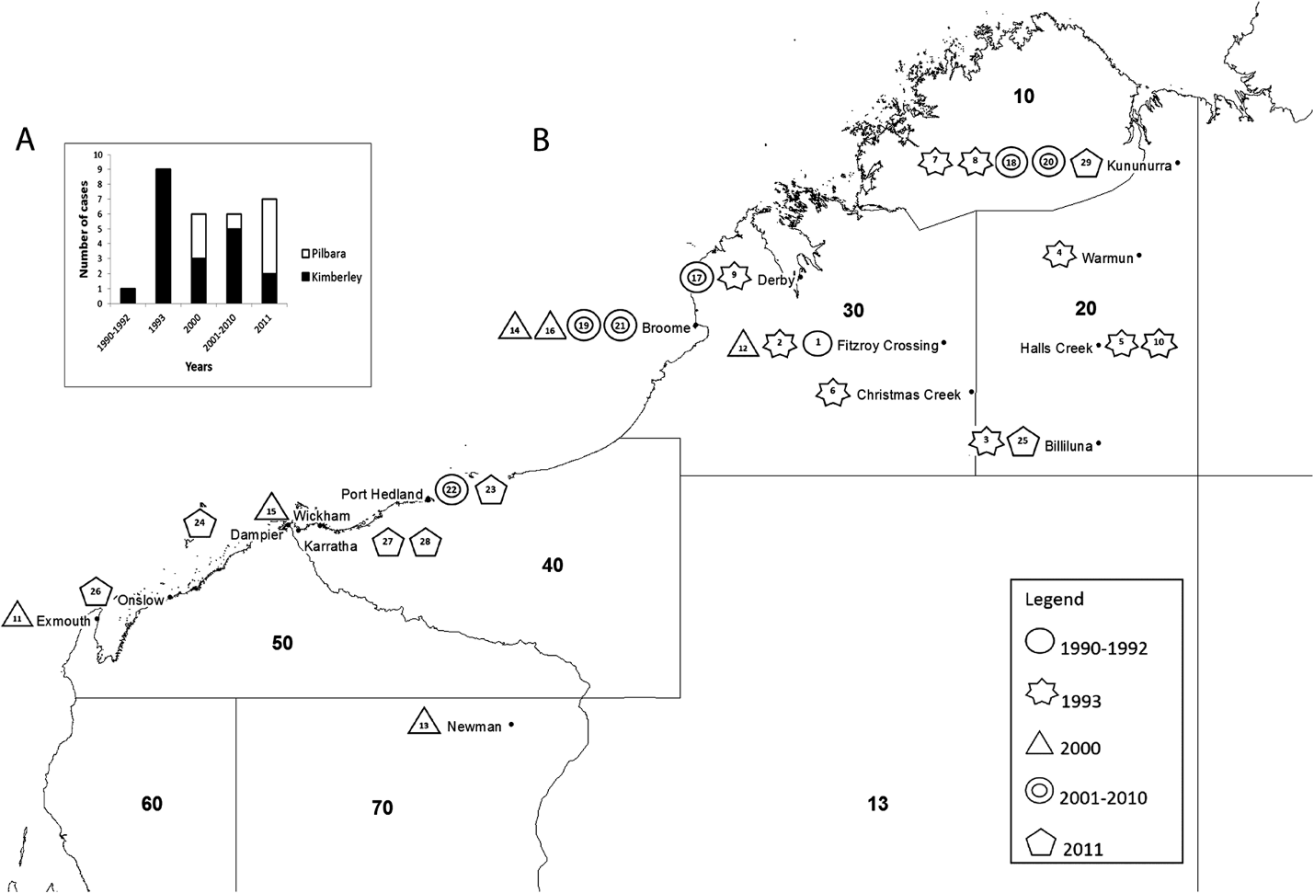
**Human cases located in Bureau of Meteorology rainfall districts and regions. A**. Location of cases by year and region. **B**. Human cases from July 1990 to Dec 2011 inclusive located in Bureau of Meteorology rainfall districts. Case numbers are in order of onset, and the shape of the symbol represents the year of onset, with three outbreak years, 1993, 2000 and 2011 indicated separately.

There were 36 human MVEV cases reported in WA in the period July 1990 – 2011 inclusive. Of these, 20 were in the Kimberley region, nine in the Pilbara, and seven in other parts of the state (in the years 2000 and 2011 only) (Figure 2). The seven cases in other parts of the state were excluded from further analysis.

### Rainfall

Rainfall was seasonal in all rainfall districts, with the peak rainfall months occurring between December and March. Monthly rainfall ranged between 0 and 608 mm in the Kimberley, and between 0 and 364 mm in the Pilbara. The highest annual rainfall in Districts 10 and 20 occurred in 2011; Districts 30 and 40 in 2000; and District 50 in 1999.

### Predictors of chicken seroconversion

Because of the large numbers of zero values for chicken seroconversion by month, the data were overdispersed and therefore the negative binomial regression model was more appropriate than the simpler Poisson regression model in all districts.

Lag rainfall two months before and the chicken seropositivity rate one month before was significantly associated with the proportion of chickens testing positive in any month in all Districts (Table 1). According to the model, for every centimetre increase in rainfall two months before (from zero), there was between a 4.3% (District 30) and 10% (District 40) increase in the chicken seroconversion for that month. Controlling for rainfall two months before and chicken seroconversion in the previous month, rainfall three months before in Districts 10, 20, 40 and 50, and rainfall one month before in District 50 was also significantly associated with chicken seroconversion. Controlling for the factors above, autumn (March-May) had the highest odds of seroconversion for Districts 30 and 40, summer (December to February) for District 20 and winter (June to August) for District 10. Season was not a significant influence on seroconversion in District 50.

**Table 1:** **Negative binomial regression models for rainfall, previous chicken seroconversion and season by rainfall district**

	District 10				District 20				District 30				District 40				District 50			
	p	Odds	95% CI		p	Odds	95% CI		p	Odds	95% CI		p	Odds	95% CI		p	Odds	95% CI	
Rain_1	NS	-	-	-	NS	-	-	-	NS	-	-	-	NS	-	-	-	<0.001	1.075	1.035	1.116
Rain_2	<0.001	1.047	1.023	1.071	.001	1.051	1.019	1.084	.001	1.041	1.016	1.066	<0.001	1.100	1.060	1.141	<0.001	1.093	1.049	1.139
Rain_3	.014	1.028	1.006	1.051	.003	1.052	1.017	1.088					.006	1.047	1.013	1.081	<0.001	1.113	1.063	1.165
Chick_1	.042	1.028	1.001	1.056	<0.001	1.043	1.024	1.063	<0.001	1.065	1.042	1.088	<0.001	1.060	1.032	1.089	<0.001	1.048	1.020	1.076
Summer	.143	2.655	.718	9.813	.013	13.466	1.750	103.634	.002	5.617	1.855	17.012	.780	.880	.358	2.161	-	-	-	-
Autumn	.364	2.003	.446	8.985	.074	7.103	.828	60.915	<0.001	13.322	4.245	41.807	.008	3.134	1.349	7.283	-	-	-	-
Winter	.029	4.316	1.163	16.023	.253	3.499	.409	29.944	.001	6.913	2.258	21.171	.029	2.487	1.098	5.632	-	-	-	-
Spring (ref)	.	1	.	.	.	1	.	.	.	1	.	.	.	1	.	.	-	-	-	-

NS = Not significant so not included in the model.

**Figure 3:**
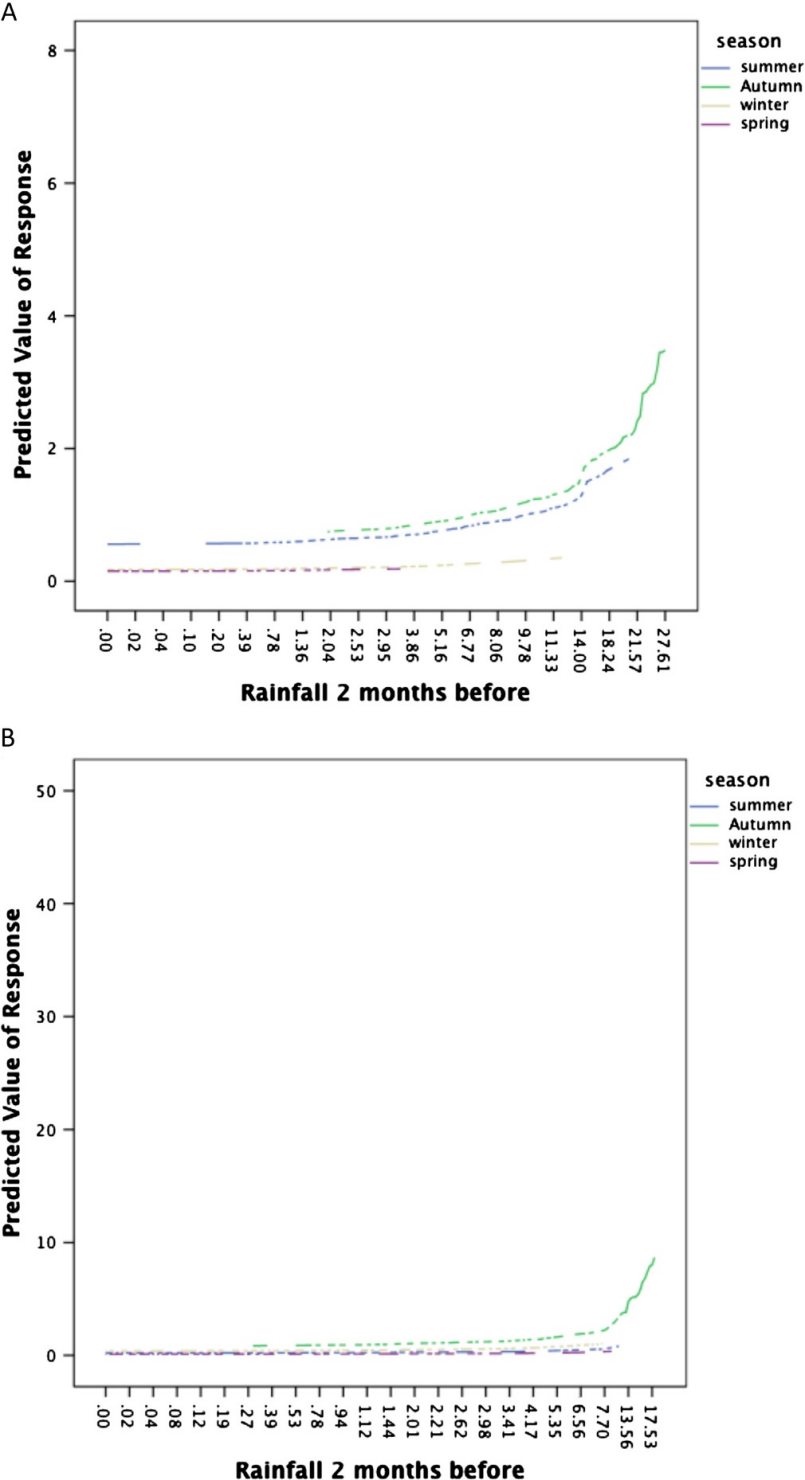
**Predictive value for the relationship between rainfall and chicken seroconversion by district.** Predictive value for the relationship between rainfall two months before and chicken seroconversion (in numbers of chickens) by district. **A**. District 20. **B**. District 50.

The predicted relationship between rainfall and seroconversion by season for districts 10 and 50 can be seen in Figure 3. To produce these graphs, the model included only rainfall two months before sampling and season, and no offset for the number of chickens being tested was used. The y-axis reflects the numbers of chickens tested in each rainfall district.

### Predictors of human cases

The small number of human cases made the negative binomial regression models unreliable (as evidenced by deviance/degrees of freedom being <0.2). However, the models were consistent with the models for the predictors of chicken seroconversion and so are shown here.

There were no human cases in District 10. Across all rainfall districts combined, rainfall two months before (Odds 1.075) and the chicken seroconversion rate one month before (Odds 1.041) were predictive of human cases (Table 2). This was also the case for District 20. Rainfall two and three months before was predictive of human cases in District 30, and rainfall one and two months before and chicken seroconversion rate one month before were predictive of human cases in the Pilbara (Table 2). Season was not significant in the models and was therefore excluded.

**Table 2:** **Predictors of human cases by rainfall district and for combined districts**

	Combined districts				District 20				District 30				Districts 40 and 50			
	p	Odds	95% CI		p	Odds	95% CI		p	Odds	95% CI		p	Odds	95% CI	
**Model 1 – Chicken seroconversion rate as predictor**																
Rain_1	NS	-	-	-	NS	-	-	-	NS	-	-	-	0.006	1.111	1.031	1.196
Rain_2	<0.001	1.075	1.048	1.102	<0.001	1.113	1.056	1.174	0.004	1.090	1.027	1.157	0.012	1.110	1.024	1.204
Rain_3	-	-	-	-	-	-	-	-	0.038	1.075	1.004	1.151	-	-	-	-
Chick_1	<0.001	1.041	1.025	1.057	0.01	1.042	1.010	1.074	-	-	-	-	<0.001	1.052	1.029	1.077
**Model 2 – Any chicken seroconversion as predictor**																
Rain_1	NS	-	-	-	NS	-	-	-	NS	-	-	-	0.002	1.112	1.041	1.189
Rain_2	<0.001	1.061	1.034	1.089	<0.001	1.127	1.071	1.187	0.001	1.102	1.039	1.168	-	-	-	-
Rain_3	-	-	-	-	-	-	-	-	-	-	-	-	-	-	-	-
AnySC_1	<0.001	10.125	3.712	27.612	-	-	-	-	0.016	13.715	1.636	114.984	0.001	13.891	2.751	70.144

NS = Not significant so not included in the model.

We transformed the ‘seroconversion’ variable into a dichotomous variable (none vs one or more seropositive chickens). Having any chicken seroconversion in the district/area of interest was predictive of human cases in that district/area one month later, with the exception of District 20, with the Odds being 10.125 for the combined Districts, 13.715 for District 30 and 13.891 for the Pilbara (Table 2). Using this variable altered the model for the Pilbara, so that rainfall two months before was not predictive of human cases, but rainfall one month before was. The ‘goodness of fit’ parameters were similar between the two models.

Sentinel chicken seroconversion pre-dated all human MVE cases except for two in March 1993 – one each in Districts 20 and 30. In March 1993, the first positive sentinel chicken was bled ten days prior to the onset of symptoms of the first human case, so that occurred during the likely incubation period for that patient. There were no sentinel chicken seroconversions anywhere in Western Australia in January and February 1993, although there were 23 seroconversions in March 1993, across Districts 10, 20 and 30. The District 20 rainfall in January 1993 (20.51 cm) was higher than the 75^th^ percentile of January rainfall (20.3 cm) and was greater than the upper 95% confidence interval of the mean of January rainfall from 1991 to 2011 (mean 17.00, 95% confidence intervals (13.80, 20.18), range 5.30 – 29.61). The District 30 rainfall in January 1993 was not significantly higher than the mean over the same period.

In March 2011, two human cases in District 40 (one in Karratha and one in Port Hedland) were preceded by chicken seroconversions in Districts 10, 20 and 50 in February 2011, but none in District 40. However, the three seroconversions in District 50 in February 2011 were located approximately 250 km from both Karratha and Port Hedland.

### Testing the model

There were no human cases in either 2012 or 2013. There were a number of chicken seroconversions in 2012 but none in 2013. Results of the model testing are shown in Additional file 1. The model testing outcomes that were inconsistent with actual findings are highlighted in yellow.

- District 10
  There were significant gaps in sentinel chicken testing in this district in 2012 and 2013, with no chickens being tested from April 2012 to April 2013 inclusive. Around 12 chickens were tested each month from May to December 2013 with one chicken seroconversion in each of January and February 2012 (25% and 20% respectively). The chicken seroconversions in January and February 2012 were consistent with the model. If sufficient chickens had been tested in May and June of 2012, the model suggests that seroconversions would have occurred. The model predicted low levels of seroconversion, if any, in 2013, consistent with the findings.
- District 20
  District 20 had five seroconversions in January (18%), three in March (14%), one in April (6%) and one in August 2012 (5%). These were consistent with the model, and the relatively low predictions for seroconversion in most of 2012 and 2013 were also consistent with the data.
- District 30
  District 30 had three seroconversions in January (7%), 11 in April (16%), five in May (9%) and two in June 2012 (4%). These were consistent with the model predictions, as were the lack of seroconversions in the other months.
- District 40
  There were four seroconversions in February (6%), 23 in March (37%), nine in April (56%) and seven in May 2012 (16%). There were no seroconversions in 2013. The seroconversions in February 2012 were above the upper confidence limit for prediction of the proportion of seroconversions for February 2012 (6% vs 2% as the upper confidence limit). The model also predicted chicken seroconversions in April 2013, even though none were observed. However, the lower confidence limit was 1%, and only 58 chickens were tested. The other outcomes were consistent with the model.
- Districts 50 and 70
  These districts had 11 seroconversions in January (24%), one in February (2%), 25 in March (27%), and four in April 2012 (29%). There were no seroconversions in 2013. The model did not predict the seroconversions in January (an upper confidence limit of 0%), or the number of seroconversions in March (an upper confidence limit of 6%). The model also predicted chicken seroconversions in March and April 2013, even though none were observed. The other outcomes were consistent with the model. The chicken seroconversions in January occurred at the Ophthalmia Dam site.

## Discussion

Our study showed that rainfall and sentinel chicken surveillance provide a useful early warning of MVEV risk to humans across endemic and epidemic areas, and our model shows that a combination of the two indicators improves the ability to assess MVEV risk and inform risk management measures.

We carried out an analysis of 22 years of sentinel chicken testing data and human cases in WA. While sentinel chickens were not consistently bled at all sites, the availability of sentinel chicken testing data from a wide geographical area and a long time-span enabled a detailed investigation of the relationship between MVEV activity and rainfall. The assessment of these as indicators of human disease was hampered by the small number of human cases over that time period, but this presented an important opportunity to evaluate the primary purpose of the surveillance system.

Rainfall in the preceding three months was consistently found to be positively related to sentinel chicken seroconversion to MVEV and human MVEV cases across the Kimberley and Pilbara regions of Western Australia. While the level of prediction may not seem great (up to 10% increase in chicken seroconversion rates for every cm increase in monthly rainfall two months before), there is considerable variability in monthly rainfall for each District from year to year. For example, in District 40, the District with the strongest relationship between lag rainfall at 2 months per cm rainfall and chicken seroconversion, the highest January rainfall for the period was almost 25 cm higher than the lowest, and the highest February rainfall was 28 cm higher than the lowest (data not shown). The relationship between human cases and rainfall was also consistently positive, with the highest being an 11.7% increase in risk in District 20 associated with each cm increase in rainfall 2 months before.

Our modelling consistently implicated rainfall as a predictor of chicken seroconversion and human cases, particularly rainfall two months prior to chicken seroconversion. This is biologically plausible, as heavy rainfall tends to cause localised flooding and filling of waterways in areas of potential MVEV activity. This in turn attracts water birds that are the recognised amplifying hosts, and enhances breeding of the known mosquito vector species [26]. Two months would allow for sufficient amplification cycles of MVEV activity in the area to result in sentinel chicken seroconversion and then human cases.

Rainfall districts in Australia are generally large areas and there can be substantial variation in rainfall and topography within districts [25]. However, there were insufficient chicken seroconversions to examine the relationship between rainfall and seroconversion for individual sentinel chicken sites, and there was a strong correlation between the mean monthly rainfall across individual sites in a rainfall district and the published monthly district rainfall.

The model appeared to be fairly robust in most rainfall districts; however it did not predict seroconversion in District 50 in January 2012, and therefore could not be used on its own to predict MVEV risk.

Other studies have investigated the association between rainfall and MVEV activity in Northern Australia. Broom et al. [26] described epizootic MVEV activity occurring in eight of 13 years in a community in the south-eastern Kimberley (District 20). They found that above average annual rainfall with extensive flooding was associated with MVEV activity, but did not always predict the level of activity. One year had below average annual rainfall but there was significant MVEV activity in that year, possibly because there was extensive flooding for part of the year, despite the low annual rainfall. With the exception of 1997, the years post 1991 in which MVEV activity was low or absent (minimum infection rates per 1000 mosquitoes less than one) despite above average rainfall with or without flooding (1994, 1997, 1999, 2001) in this community [26], were years that we found had above average chicken seroconversion rates across District 20 (data not shown). This suggests that rainfall levels predict MVEV activity in a broad area, but other factors at local sites will affect the local risk.

Interestingly, Schuster et al. [27], using satellite data analysis to estimate rainfall at a local level found an association between rainfall two months prior and any MVEV activity in the Pilbara and rainfall three months prior and any MVEV activity in the Kimberley between March 2000 and December 2007 [27]. However, while these results are consistent with our findings for the Districts 10, 20, 40 and 50, they did not match ours for District 30, as we did not find an association between rainfall three months prior and MVEV activity in that district. Our models included data over a longer period of time and across more than one sentinel chicken site, enabling the inclusion of other factors in the analysis. We also didn’t assess absolute MVEV activity (Y/N), but rather levels of MVEV activity as measured by seroconversion rates, and therefore it is not surprising that our results were not completely consistent. Satellite data is difficult and can be expensive to obtain, so the Bureau of Meteorology rainfall data offers a more practical ongoing source of rainfall data.

While we found an association between rainfall and MVEV activity, we did not identify a consistent rainfall cut-off that would predict MVEV activity in WA. This contrasts with a report from Central Australia showing that MVEV chicken seroconversion and human MVEV disease only occurred in years when the annual rainfall was greater than 300 mm and the summer (December to February) rainfall was greater than 100 mm [16]. As these cases occurred in an epizootic areas following extensive flooding, the association between rainfall and disease may be more consistent than we found in enzootic and/or more climatically diverse areas of WA.

In addition we showed that rainfall is associated with the lower levels of MVEV activity that are associated with sporadic human cases. These cases occur in endemic areas where the relationship between rainfall, MVEV activity and human risk is expected to be less predictable than in outbreak situations.

Sentinel chickens have been used widely to improve the assessment of human risk of flavivirus disease. Studies in the USA and elsewhere have found that sentinel chicken seroconversions to West Nile virus and St Louis encephalitis virus had varying efficacy in predicting the onset of human cases [20],[28]-[31]. In many instances, sentinel chickens have been more useful in rural settings than in urban settings [32],[33], probably due to vector species and vector dispersal, and to various environmental factors. This is supported by a modelling study that suggested that the efficacy of sentinel animals in predicting human cases is dependent on the vector infection rate, the biting rate on the target animals and the number of sentinel animals used, so it cannot be assumed that the success of sentinel chickens in one situation will translate to another [20]. Thus the site for maintaining sentinel chicken flocks is a critical aspect in their use in surveillance. Furthermore even relatively low levels of virus activity may result in large numbers of human cases when the activity occurs in the vicinity of large populations [20],[28]-[31].

With the exception of March 1993, chicken seroconversion preceded any human cases in WA by at least one month. However, in March 1993, the chicken seroconversions preceded the first human case by less than 10 days, even though there was consistent testing of sentinel chickens in the two months prior. In spite of consistent testing, the time taken for chicken seroconversion following infection (approximately 5 to 10 days) means that there are inherent delays in detecting infection in sentinel chickens. When the seroconversions occurred in the same month as the human cases, this is unlikely to give sufficient time to provide warnings to prevent the initial human cases. However, if rainfall two months prior to the onset of the human case had been taken into account for assessing human risk, it is likely that the human cases would have been foreseen.

MVEV is thought to be maintained in a few enzootic sites in north-west WA (predominantly in District 20), and following sufficient rainfall and movement of waterbirds, it is spread to other parts of the state and adjacent areas of the Northern Territory [3],[26]. Consistent with this, waterbird movement from the Ord River region of the Kimberley to the South Eastern Kimberley has been observed in wet years [26]. In some years (1998, 2005, 2007), however, MVEV activity, as evidenced by sentinel chicken seroconversions, occurred simultaneously in both enzootic and epizootic areas. While this may reflect rapid movement of viraemic birds from enzootic to epizootic areas due to favourable conditions [26], we cannot exclude the possibility that there may be unrecognised enzootic foci of MVEV within the epizootic areas that are then amplified by waterbirds, as has been seen with the tick-borne flaviviruses [34]. Also the survival of virus through the dry season in desiccation-resistant eggs of *Aedes* mosquito species has been proposed previously as a source of MVEV activity in the south-east Kimberley [3], and may possibly occur in other areas.

In the Kimberley, Lake Argyle, which is the largest man-made lake in Australia (5641 gigalitres), and a diversion dam, both situated near Kununurra in District 20, were constructed in the 1960’s and 1970’s [35]. Both of these dams are part of the Ord River Irrigation Scheme and the diversion dam area, including the town of Kununurra and its surrounding irrigation area, has been recognised as an extensive mosquito breeding site and habitat for birds for many years [35]. These developments have resulted in profound ecological changes including greatly enhanced conditions for mosquito-borne virus transmission, and the potential to assist the spread of virus activity elsewhere in the region [36]. Similarly, construction in the 1980’s of Harding Dam (District 40) and Ophthalmia Dam (District 70) in the Pilbara has created unique large permanent water sites that could maintain mosquito breeding and bird habitats during times of lower rainfall. This may account for the high number of chicken seroconversions in January 2012 that occurred at Ophthalmia Dam but were not predicted by our model. It is not clear whether these dams may act as initiating sites for MVEV activity in the Pilbara as neither of the dams was consistently the first site for MVEV activity in that region. Unfortunately, in some years there was insufficient chicken testing at Ophthalmia and Harding Dams to know whether or not there was MVEV activity at these sites prior to activity being detected elsewhere. Further research is required to elucidate the situation.

It is clear that the relationship between rainfall, sentinel chicken seroconversions and human disease risk is complex and influenced by a number of factors including whether MVEV activity is in an endemic/enzootic or an epidemic/epizootic area, the placement of the sentinel chicken flocks, the proximity of the MVEV activity to human populations and the individual risk behaviour [15]. However, it is also clear that the combination of rainfall monitoring and sentinel chicken surveillance provides the best estimate of human risk currently available. Due to the remoteness of MVEV activity in WA, having reliable sentinel chicken bleeding can be challenging and regular mosquito trapping is not a logistically feasible option. However, recent studies on the use of non-powered CO_2_-baited traps and honey-soaked cards to capture viral nucleic acid from mosquito saliva has been shown to be more sensitive than sentinel chickens in detecting WNV_KUN_ in Northern Australia, and may, therefore, provide an effective and more practical system for detecting MVEV activity in the future, although further work on the passive traps is required [37]-[40]. Use of this system in WA could then be assessed against sentinel chicken data for its value in predicting human risk.

Since 1998, the WA Department of Health has implemented a contingency plan to ensure a consistent approach to public health protection measures following detection of seroconversion to MVEV in sentinel chickens. Under the plan, public warnings and mosquito management programs are implemented where practicable across entire meteorological districts in response to detection of MVEV activity, not just at the locality of the positive chicken flock. The results of this study support a continuation of that regional interpretation and management response to localised sentinel chicken seroconversions.

## Conclusions

Rainfall and sentinel chicken surveillance provide a useful early warning of MVEV risk to humans across endemic and epidemic areas in northern Western Australia, and our modelling shows that a combination of the two indicators improves the ability to assess MVEV risk and inform risk management measures.

## Additional file

## Electronic supplementary material

Additional file 1: This shows the outcomes of testing the model by rainfall district. (XLSX 16 KB)

Below are the links to the authors’ original submitted files for images.

Authors’ original file for figure 1

Authors’ original file for figure 2

Authors’ original file for figure 3

## Abbreviations

MVEV: Murray Valley encephalitis virus
WA: Western Australia
WNV_KUN_: Kunjin strain of West Nile virus
PathWest: PathWest Laboratory Medicine WA, QEII Medical Centre
BOM: Bureau of meteorology
