# Supplementary figures and images for: Rainfall and sentinel chicken seroconversions predict human cases of Murray Valley encephalitis in the north of Western Australia
Source: BMC Infect Dis. 2014 Dec 10;14:672. doi: 10.1186/s12879-014-0672-3 (PMC4273426; doi:10.1186/s12879-014-0672-3)

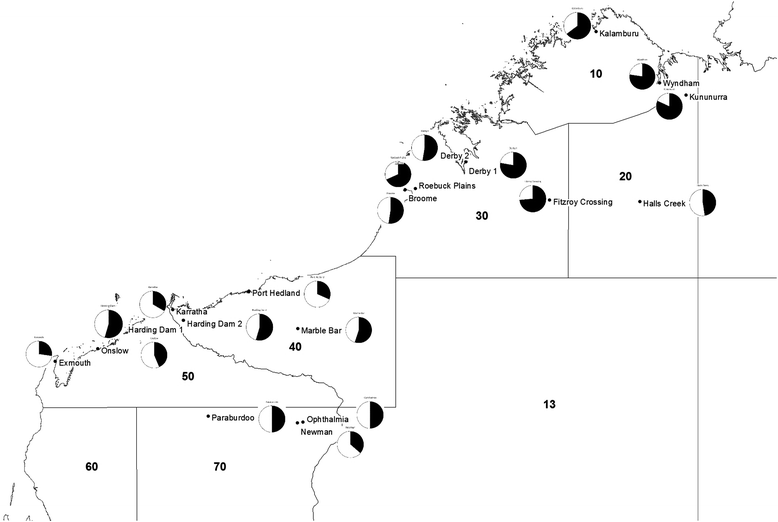

Supplement: Supplementary file 2 — Authors’ original file for figure 1 [file 12879_2014_672_MOESM2_ESM.gif]

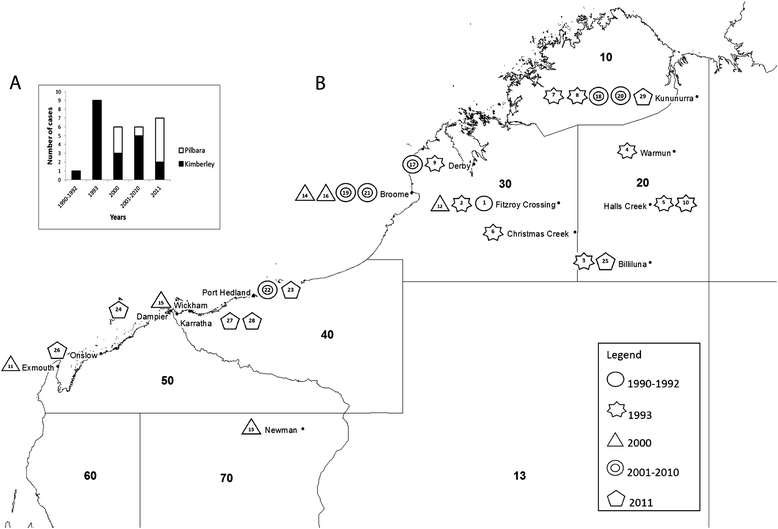

Supplement: Supplementary file 3 — Authors’ original file for figure 2 [file 12879_2014_672_MOESM3_ESM.gif]

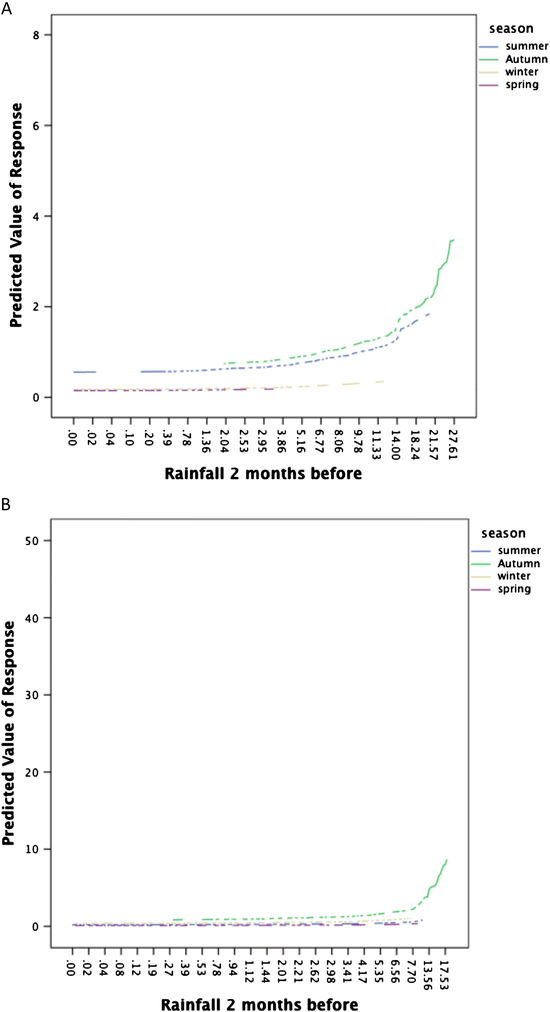

Supplement: Supplementary file 4 — Authors’ original file for figure 3 [file 12879_2014_672_MOESM4_ESM.gif]
